# Injectable biocompatible nanocomposites of Prussian blue nanoparticles and bacterial cellulose as a safe and effective photothermal cancer therapy

**DOI:** 10.1186/s12951-023-02108-6

**Published:** 2023-10-05

**Authors:** Hwichan Hong, MinKyu Kim, Wooseung Lee, Miyeon Jeon, Chaedong Lee, Hoonsub Kim, Hyung-Jun Im, Yuanzhe Piao

**Affiliations:** 1 Department of Applied Bioengineering, Graduate School of Convergence Science and Technology, Seoul National University, Seoul, 08826 Republic of Korea; 2 Department of Molecular Medicine and Biopharmaceutical Sciences, Graduate School of Convergence Science and Technology, Seoul National University, Seoul, 08826 Republic of Korea; 3 Cancer Research Institute, Seoul National University, Seoul, 03080 Republic of Korea; 4 Advanced Institutes of Convergence Technology, Seoul National University, Suwon-si, Gyeonggi-do Republic of Korea; 5 Research Institute for Convergence Science, Seoul National University, Seoul, Republic of Korea

**Keywords:** Prussian blue nanoparticles, Bacterial celluloses, Nanocomposites, Photothermal therapy, Improved biocompatibility

## Abstract

Photothermal therapy (PTT) is a novel cancer treatment using a photoabsorber to cause hyperthermia to kill tumors by laser irradiation. Prussian blue nanoparticles (PB NPs) are considered as next-generation photothermal agents due to the facile synthesis and excellent absorption of near-infrared light. Although PB NPs demonstrate remarkable PTT capabilities, their clinical application is limited due to their systemic toxicity. Bacterial cellulose (BC) has been applied to various bio-applications based on its unique properties and biocompatibility. Herein, we design composites with PB NPs and BC as an injectable, highly biocompatible PTT agent (IBC-PB composites). Injectable bacterial cellulose (IBC) is produced through the trituration of BC, with PB NPs synthesized on the IBC surface to prepare IBC-PB composites. IBC-PB composites show in vitro and in vivo photothermal therapeutic effects similar to those of PB NPs but with significantly greater biocompatibility. Specifically, in vitro therapeutic index of IBC-PB composites is 26.5-fold higher than that of PB NPs. Furthermore, unlike PB NPs, IBC-PB composites exhibit no overt toxicity in mice as assessed by blood biochemical analysis and histological images. Hence, it is worth pursuing further research and development of IBC-PB composites as they hold promise as safe and efficacious PTT agents for clinical application.

**Graphical Abstract:**

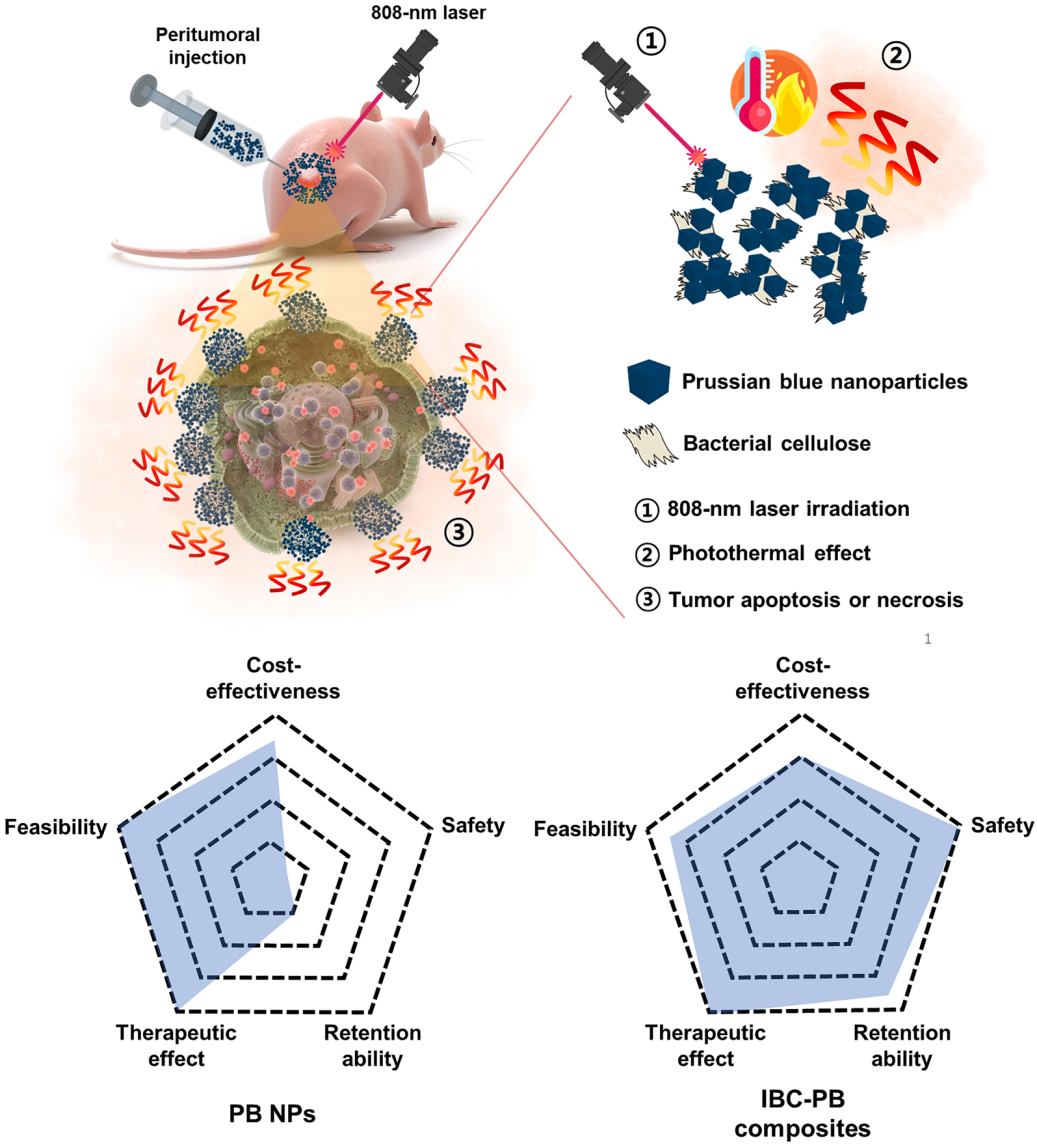

**Supplementary Information:**

The online version contains supplementary material available at 10.1186/s12951-023-02108-6.

## Introduction

Photothermal therapy (PTT) is an emerging cancer treatment strategy that has been extensively researched in recent years [1–4]. This method involves the use of near-infrared (NIR) laser to irradiate photothermal agents accumulated in cancer lesions. These agents then convert the absorbed light into heat, causing a rise in local tissue temperature and thereby causing apoptosis or necrosis of tumor cells [5–8]. Numerous materials, including carbon-based materials [9, 10], gold [11–13], silver [14–16], iron oxide [17, 18], and germanium nanocrystals [19, 20], are currently being developed as potential agents for PTT. Also, a recent phase 1 clinical trial in patients with prostate cancer demonstrated the feasibility of PTT using a gold nanoshell, with successful tumor ablation and no serious complications observed in 94% (15/16) of patients [21]. This study demonstrates the promise of PTT agents for clinical applications, but the high cost of gold nanoparticles is considered as a potential limitation hindering large-scale clinical trials [22].

Prussian blue (PB) is an ancient low-cost dye that can be easily prepared [23]. Due to its strong optical absorbance in the NIR region and good photostability, Prussian blue nanoparticles (PB NPs) have been developed as potential photothermal agents for cancer [24–26]. PB NPs have gained significant attention in the biomedical field primarily due to their facile synthesis, which allows for rapid and efficient production. Their controllable size and shape, coupled with the ease of surface modification, offer multifunctionality, making them ideal for a range of applications like theranostics and drug delivery. Additionally, their cost-effectiveness ensures that they remain an attractive option for researchers and practitioners looking for budget-friendly yet effective solutions [27–30]. In practice, the United States Food and Drug Administration (US FDA) approved PB NPs as a clinical medicine for oral administration to treat patients with internal contamination of thallium (Tl^+^) or radiocesium (Cs^+^) poisoning in 2003 [30]. However, PB NPs have disadvantages as well, such as the accumulation in normal organs when they are systemically administered [31]. In particular, Chen et al. reported that intravenously injected PB NPs in mice caused acute liver injury, an outcome attributed to the substantial accumulation of the nanoparticles in Kupffer cells, the resident macrophages in the liver [32].

Bacterial cellulose (BC) stands out in the realm of biomaterials due to its exceptional biocompatibility, allowing it to be used in biological systems without causing adverse reactions. BC’s inherent flexibility means it can conform to various shapes and structures, making it especially useful in situations that demand adaptability, such as molds or dynamic biomedical environments. The porous nature of BC not only provides it with a lightweight structure but also allows for efficient delivery and retention of bioactive agents, further establishing its potential in drug delivery systems and tissue engineering scaffolds. Based on these properties, BC is used in various biomedical applications, such as diagnostic sensors [33, 34], tissue engineering [35], drug delivery systems [36–38], wound dressing [39–42], and artificial skin [43]. Nata de coco, a type of BC invented in 1949, is made by fermenting coconut water through Komagataeibacter xylinus [44]. Bacterial cellulose can be used by itself, but it is also used to make composites with various additives such as biopolymers [45, 46], quantum dots [47, 48], nanoparticles [39, 40, 49, 50] and nano carbons [51, 52]. Nanocellulose reportedly has an excellent stabilizing effect for metal nanoparticles, which can promote the nucleation of nanoparticles as well as prevent their agglomeration [53]. Therefore, we hypothesized that BC could be used to stabilize PB and reduce its the toxicity.

Herein, we successfully manufactured and synthesized a composite of injectable bacterial cellulose with PB NPs that is biocompatible, locally injectable, and is amenable with repeated PTT sessions. The injectable BC composite with PB NPs (IBC-PB composites) went through a simple reduction reaction. The PB NPs were directly grown onto BC fragmented on the nanoscale. We hypothesized that IBC-PB composites can exert a potent PTT effect while remaining less toxic compared to PB NPs due to the biocompatibility of BC and the enhanced stability realized here.

## Results and discussion

### Injectable bacterial cellulose (IBC) synthesis from nata de coco

Nata de coco is purified by flowing deionized water (D.W.) for several days to remove impurities such as remaining bacteria and sugar. After purification, physical grinding and lyophilization were conducted for an accurate quantitative experiment. Schweitzer’s reagent was used to reduce the nanofiber structure of BC to obtain an injectable bacterial cellulose. After the pre-treatment, the injectable quantified bacterial cellulose was successfully obtained (Additional file 1: Fig S1).

### Synthesis of PB NPs and IBC-PB composites

**Fig. 1:**
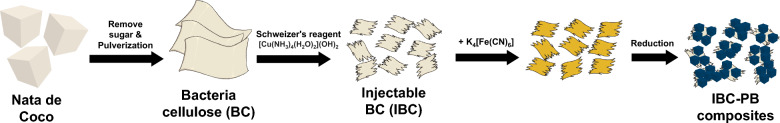
Schematic diagram of the IBC-PB composites synthesis procedure

For the PB and IBC-PB composites, they were simply synthesized by the reduction of K_3_[Fe(CN)_6_] under an acidic condition at 80 ℃ with the addition of Polyvinylpyrrolidone (PVP) or IBC. The synthesized IBC-PB composites were dispersed at 50 ml D.W. and kept in a refrigerator at 5 ℃ to prevent contamination (Fig. 1).

### Characterizations of the PB NPs and IBC-PB composites

**Fig. 2:**
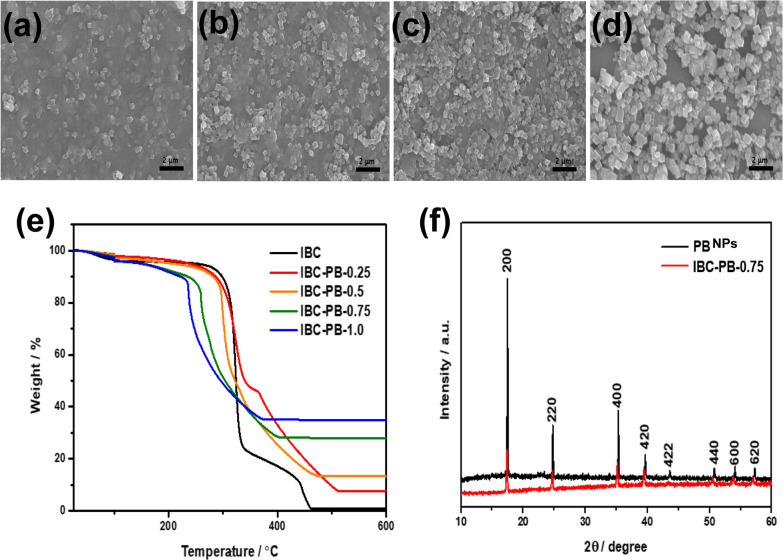
Characterization of the IBC-PB composites: (a-d) SEM images of an IBC-PB composites according to the K_4_[Fe(CN)_6_] concentration gradient (left to right: (**a**) 0.25, (**b**) 0.5, (**c**) 0.75, and (**d**) 1 mmol of K_4_[Fe(CN)_6_)]. **e** Thermogravimetric analysis (TGA) of an IBC-PB composites according to the K_4_[Fe(CN)_6_] concentration gradient (0, 0.25, 0.5, 0.75, and 1 mmol of K_4_[Fe(CN)_6_)]. **f** X-ray diffraction (XRD) pattern data of PB NPs and IBC-PB composites. (*IBC-PB* IBC-PB composites)

The morphology of IBC-PB composites were examined by scanning electron microscope (SEM), and PB NPs with a cubic structure were found to be evenly distributed on the IBC sheet (Fig. 2a–d). The ratio of PB NPs per area and particle size increases according to the concentration of potassium ferricyanide (0.25, 0.5, 0.75, and 1 mmol respectively). The PB NP size of the IBC-PB composites were 150–275 nm, and we synthesized 200 nm PB NPs as a control (Additional file 1: Fig S2). Pristine PB NPs were utilized as a control in all further in vitro and in vivo studies.

TGA analysis was conducted under an air condition to measure the volume of PB nanoparticles (Fig. 2e). From the results of the TGA analysis of BC, only 0.8% of the current weight of cellulose remained after calcination at a temperature that exceeded 600 ℃, implying that all cellulose was removed at this high temperature. According to this result, the remaining important material of IBC-PB composites after TGA was confirmed to be iron oxide, which was produced by the calcination of PB, with the level proportional to the amount of potassium ferricyanide from 0 to 1 mmol.

The XRD patterns of PB and IBC-PB composites are matched with that of a face-centered cubic lattice and are in good agreement with the standard data of PB (ICDD PDF2 01-073-0687). The peaks at 2-theta values of 17.4, 24.7, 35.1, 39,4, 43.5, 50.8, 54.1 and 57.3° can be assigned correspondingly to the (200), (220), (400), (420), (422), (440), (600) and (620) planes of the PB (Fig. 2f).

The FT-IR spectroscopy displayed a characteristic peak of CN stretching at 2085 cm^−1^, O-H around 3300 cm^−1^, C-O-C stretches at 1050 m^−1^ (Additional file 1: Fig S3).

SEM, XRD, TGA, FT-IR data showed that Prussian blue nanoparticles were synthesized proportionally to the amount of the initial PB precursor and were uniformly distributed on the BC sheet.

### Photothermal effect of the IBC-PB composites

**Fig. 3:**
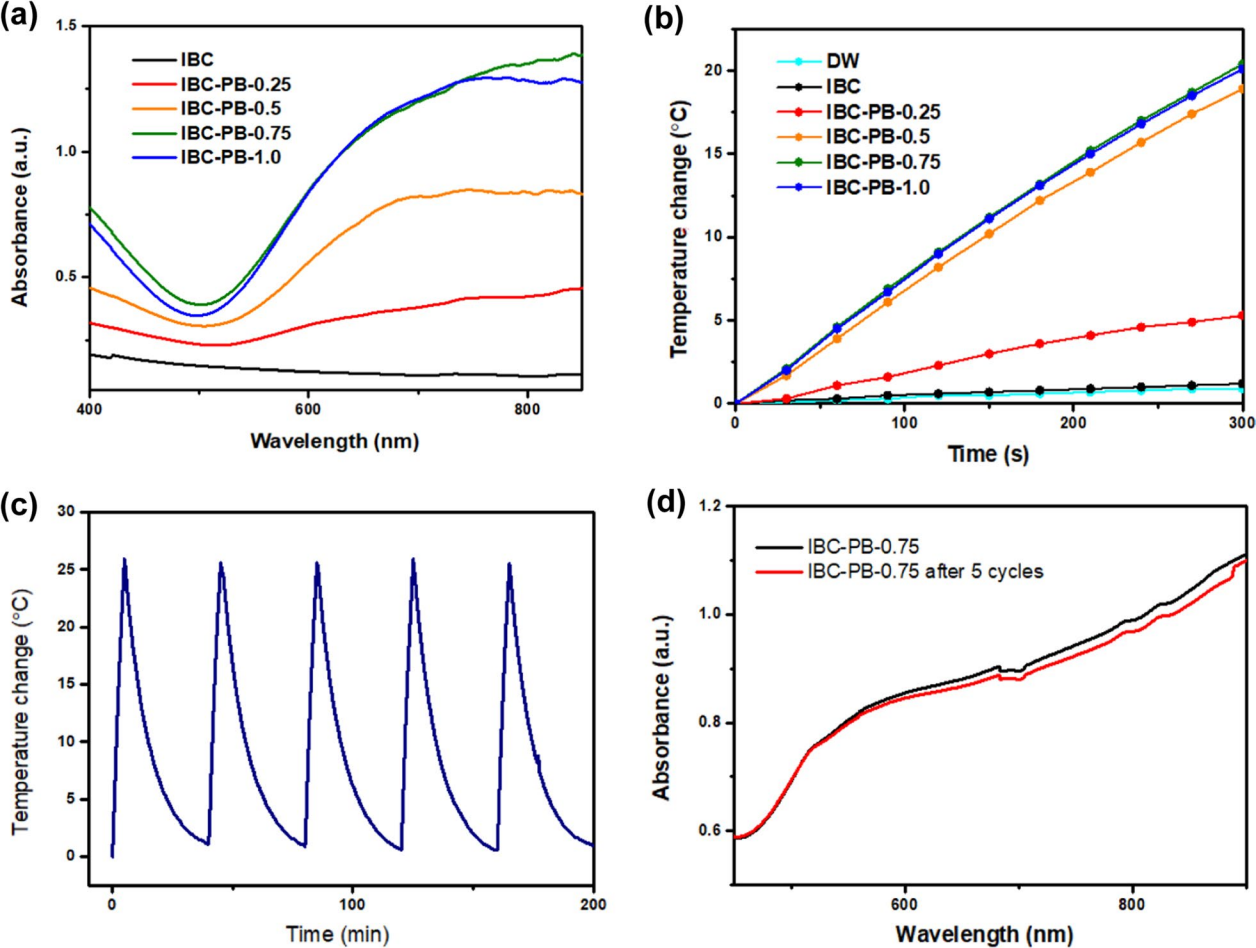
Photothermal characterization of the IBC-PB composites: (**a**) absorbance spectra scanning and (**b**) photothermal effect of the IBC-PB composites in the series of K_4_[Fe(CN)_6_] concentrations (0, 0.25, 0.5, 0.75, and 1 mmol of K_4_[Fe(CN)_6_] at an intensity level of 1 W cm^−2^ for five minutes. **c** Validation of the photothermal ability of the IBC-PB composites during five cycles of laser irradiation at an intensity level of 1 W cm^−2^ for 200 min. **d** Absorbance spectra comparison of IBC-PB composites with 0.75 mmol of K_4_[Fe(CN)_6_] before and after five repeated laser irradiation cycles. (*IBC-PB* IBC-PB composites)

The absorbance spectra of the IBC-PB composites according to a UV–visible spectrometer showed absorption bands at 600–900 nm with a high peak value at 800–900 nm. Due to its strong NIR absorption, IBC-PB composites had noticeable photothermal effects. The absorbance intensity increased in a linearly proportional manner relative to the initial concentration of potassium ferricyanide from 0.25 to 0.75 mmol. The absorbance of the IBC-PB composites (1 mmol) was mostly in accord with IBC-PB composites (0.75 mmol), allowing us to confirm that saturation accrued at 0.75 mmol (Fig. 3a).

During the photothermal test, the temperature of the IBC-PB composites in each case was increased by irradiation using an 808 nm-laser with at an intensity level of 1 W cm^−2^ by 20.5 ℃ in 5 min (Fig. 3b). The photothermal effect of IBC-PB composites increased with the amount of PB from 0.25 to 0.75 mmol, with the results for IBC-PB composites (1 mmol) in good agreement with those of IBC-PB composites (0.75 mmol). This is consistent with the UV–vis spectra data, where the photothermal effect was saturated at 0.75 mmol of K_4_[Fe(CN)_6_]. Therefore, IBC-PB composites (0.75 mmol) was selected as the leading group for all further in vitro and in vivo studies.

### Repetitive photothermal ability of IBC-PB composites

In the repetition demonstration, the laser was irradiated five times for five minutes and the temperature rise was maintained by 25 ℃. As a result, the photothermal effect of IBC-PB composites was maintained after multiple laser irradiation trials (Fig. 3c). After five cycles, the UV–vis spectra confirmed that the absorbance at NIR was maintained (Fig. 3d).

### In vitro photothermal therapeutic effect in the 4T1 breast cancer cell line

**Fig. 4:**
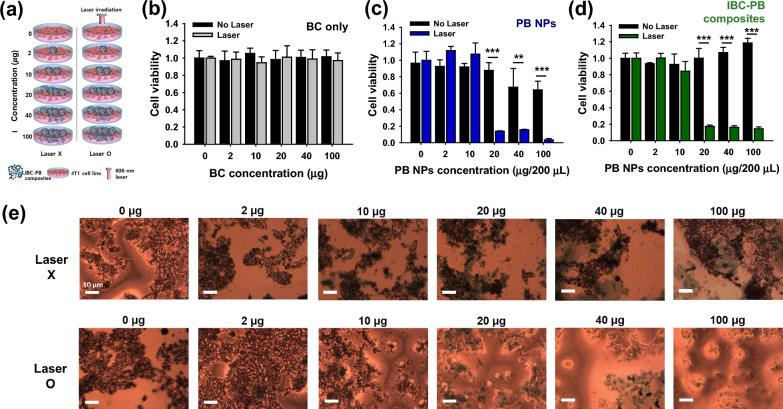
In vitro PTT effect study: (**a**) experimental design of in vitro PTT in the 4T1 breast cancer cell line. In vitro PTT effect (808 nm, 2 W cm^−2^, 5 min) according to the concentration gradient (0, 2, 10, 20, 40, 100 μg 200 μL^−1^) of (**b**) IBC, (**c**) PB NPs, and (**d**) IBC-PB composites (n = 3, mean ± s.d.). **e** Optical images of cell populations treated with the series of IBC-PB composites with and without PTT. *: P < 0.05, **: P < 0.01, ***: P < 0.001. The statistical analysis was conducted by means of student’s t test for b, c, and d

The in vitro PTT effects of the BC, PB NPs and IBC-PB composites were evaluated using an 808 nm NIR laser (Fig. 4a). BC had no overt cytotoxicity with or without laser irradiation (Fig. 4b). The unirradiated IBC-PB composites showed no cytotoxicity at all treated concentrations. The laser-irradiated IBC-PB composites showed a dramatic photothermal therapeutic effect with more than 82% cell killing ability from a PB concentration of 20 μg 200 μL^−1^ (Fig. 4d). Live 4T1 cells were scarcely observed after in vitro PTT at PB concentrations above 20 μg 200 μL^−1^ in the IBC-PB composites. However, the cells were intact in the IBC-PB composites without PTT (Fig. 4e). On the other hand, despite the fact that the PB NPs displayed a high PTT effect of more than 80% cell death when irradiated by an 808 nm laser at a concentration of 20 μg 200 μL^−1^, the unirradiated PB NPs also exhibited increased cytotoxicity as their concentration was increased. This suggests that cell death was caused not only by the PTT effect but also by the inherent cytotoxicity of the PB NPs (Fig. 4c).

### In vivo retention at the tumor region of PB NPs and IBC-PB composites

**Fig. 5:**
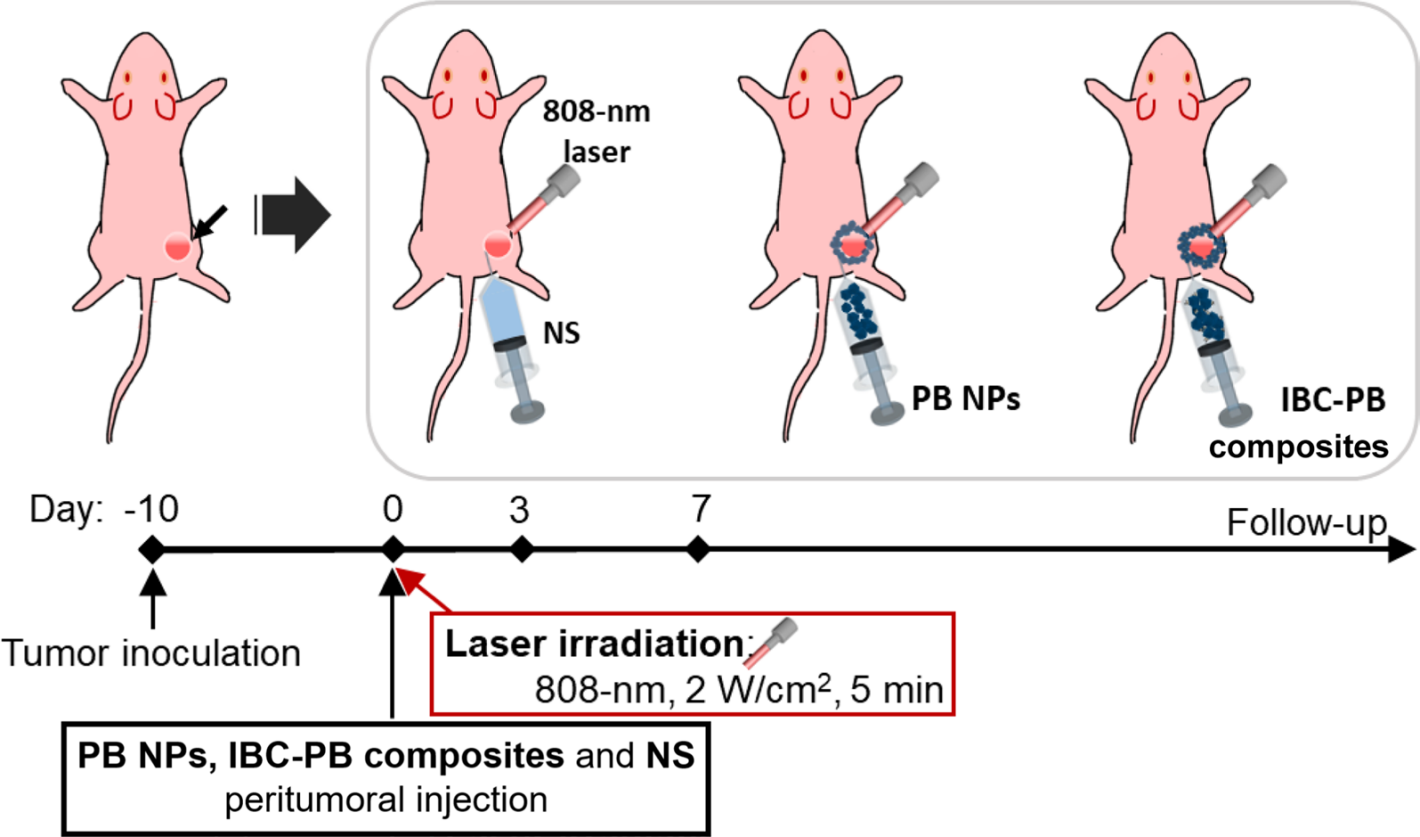
Experimental design of IBC-PB composites-mediated in vivo PTT

We also conducted an in vivo PTT experiment with 4T1 tumor-bearing mice. We injected PB NPs and IBC-PB composites peritumorally as earlier studies reported that doing so resulted in better positioning and an improved PTT effect compared to an intratumoral injection [30, 56]. We compared the retention profiles of the PB NPs and the IBC-PB composites after a peritumoral injection around the subcutaneously located 4T1 tumor tissue region with 4T1 tumor-bearing mice (n = 3) for seven days (Additional file 1: Fig S4). The peritumoral injections of the PB NPs and IBC-PB composites were clearly visible around the tumor region immediately after the injection (day 0). However, the PB NPs rapidly disappeared and were difficult to detect 24 h post-injection due to systemic absorption and were not visible in the resected tumor images seven days after the injection. In contrast, the IBC-PB composites remained at the site of the injection up to seven days post-injection, as evidenced by images taken of resected tumors. It should be noted that the IBC-PB composites were not fully absorbed by the body, suggesting that the IBC-PB composites could be repeatedly targeted by an 808 nm laser for complete tumor removal via repeatable PTT and that they can also be considered more biocompatible than PB NPs. Peritumoral injection provides a notable advantage by improving the precise delivery of treatment to the tumor location, while reducing the risk of adverse effects on the entire body [54–56]. Furthermore, the combination of this method with the immunotherapy shows potential for positive outcomes, even when dealing with metastatic cancers (Fig. 5) [57, 58].

### In vivo photothermal imaging and therapy in 4T1 cancer-bearing mice

**Fig. 6:**
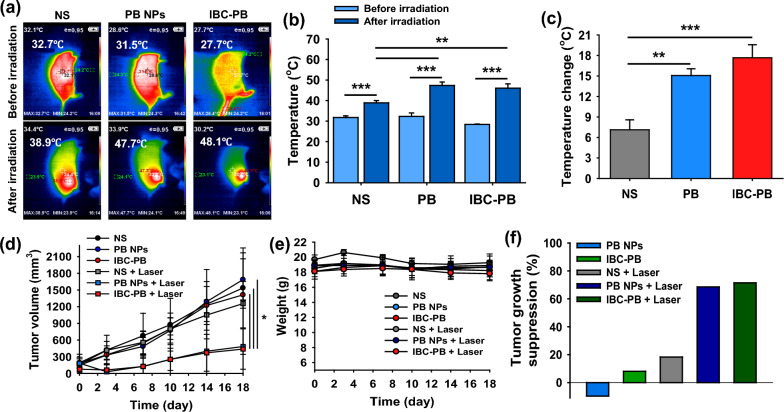
In vivo photothermal imaging and PTT study: **a** in vivo photothermal imaging with NS, PB NPs, and IBC-PB composites in a 4T1 tumor bearing Balb/c nude mouse model. **b** Temperature changes in the tumor regions after laser irradiation (808-nm, 2 W cm^−2^, 5 min) of NS, PB NPs, and IBC-PB composites (n = 3, mean ± s.d.). **c** Temperature increases at the tumor regions before and after laser irradiation of NS, PB NPs, and IBC-PB composites (n = 3, mean ± s.d.). **d** Tumor volume and (**e**) body weight profiles of 4T1 tumor-bearing mice after PTT (808 nm, 2 W cm^−2^, 5 min) peritumorally injected with NS, PB NPs, and IBC-PB composites (n = 3, mean ± s.d.). **f** Tumor growth suppression ratio 18 days after follow-up based on tumor volume measurements. *: P < 0.05, **: P < 0.01, ***: P < 0.001. The statistical analysis was conducted by means of a student’s t test for b, c, and d. (*IBC-PB* IBC-PB composites)

For the in vivo photothermal imaging and therapy, normal saline (NS), PB NPs, and IBC-PB composites were peritumorally injected into 4T1 tumor bearing mice. After the injection in each group, an 808 nm NIR laser was irradiated under the condition of 2 W cm^−2^ for five minutes only once. The three different injected materials were separated into two groups, referred to the laser irradiated and unirradiated groups (NS, PB NPs, IBC-PB composites, NS + Laser, PB NPs + Laser, and IBC-PB composites + Laser). In vivo photothermal imaging was conducted and the temperature distribution at the tumor sites was observed (Fig. 6a). The temperature at the NS-injected tumor area increased by only 6.2 °C from 32.7 to 38.9 °C after 808-nm laser irradiation. In contrast, the temperature in the tumors injected with PB NPs and IBC-PB composites increased dramatically, reaching 47.7 °C (ΔT: 16.2 °C) and 48.1 °C (ΔT: 20.4 °C), respectively. The IBC-PB composites showed a slightly larger temperature change of 4.2 °C compared to that of the PB NPs (Fig. 6b, c). This demonstrates that the IBC-PB composites has a potent photothermal effect in vivo.

The tumor tissue growth in the unirradiated groups and the NS + laser group increased proportionally with time. In contrast, the PB + Laser and IBC-PB composites + Laser groups showed significantly smaller tumor sizes compared to the control groups (NS, PB NPs, and IBC-PB composites) (P < 0.05 for all comparisons at day 18) (Fig. 6d). The suppressed tumor growth was evaluated by examining the tumor volume up to 18 days (Fig. 6e). The IBC-PB composites showed a 71.5% tumor growth suppression rate, demonstrating greater tumor-growth inhibition ability compared to the control groups, particularly the PB NPs (68.5% tumor-growth suppression effect). The body weight up to 18 days after treatment was not significantly reduced in all groups (Fig. 6f).

### In vitro cytotoxicity test in the RAW 264.7 macrophage cell line

**Fig. 7:**
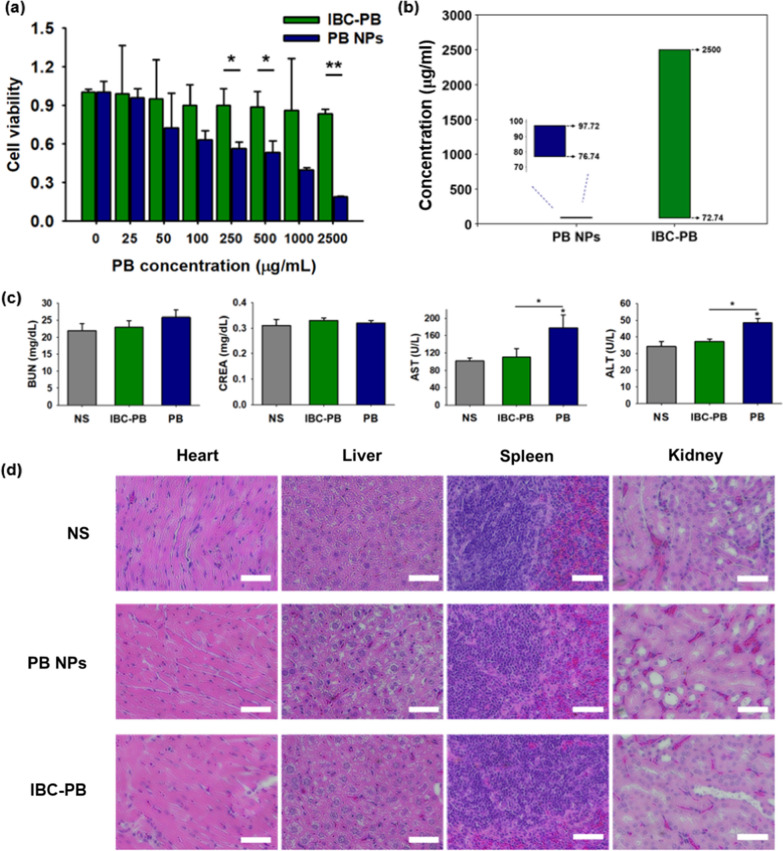
Cytotoxicity and systemic toxicity effect of the IBC-PB composites: (**a**) in vitro cytotoxic effect comparison between the IBC-PB composites and the PB NPs in the RAW 264.7 cell line (n = 3, mean ± s.d.). **b** In vitro therapeutic window of the PB NPs and IBC-PB composites. **c** Biochemical analysis of blood 24 h after a subcutaneous injection of the NS, PB NPs, and IBC-PB composites into mice (kidney function indices: blood urea nitrogen (BUN) and creatine (CREA), liver function indices: aspartate transaminase (AST) and alanine transaminase (ALT)) (n = 3, mean ± s.d.). **d** H&E staining paraffin-sectioned images of major organs 24 h after a subcutaneous injection of NS, PB NPs, and IBC-PB composites in mice. *: P < 0.05, **: P < 0.01, ***: P < 0.001. The statistical analysis was conducted by means of a student’s t test for a and b. (IBC-PB: IBC-PB composites)

Given the limitations in the toxicity of PB NPs, we undertook a further evaluation of the toxicity profiles of both the PB NPs and the IBC-PB composites. To do this, we conducted an MTT assay using RAW 264.7 macrophage cells (Fig. 7a). The PB NPs demonstrated an increasing toxic effect as the PB concentration was increased. The PB NPs started to show cell viability of less than 72% at a PB concentration of 50 μg mL^−1^ and showed cell viability of 19% at a concentration of 2500 μg mL^−1^. Meanwhile, the IBC-PB composites showed no noticeable cytotoxic effect up to 2500 μg mL^−1^, and the viability of IBC-PB composites-treated cells was 4.4-fold higher than that of PB NPs-treated cells at that concentration. It was clear that IBC-PB composites were far less toxic than PB NPs in RAW 264.7 macrophage cells. We also measured the in vitro therapeutic window (Fig. 7b) based on the cell viability presented in Figs. 4c, d, 7a. We defined the in vitro therapeutic window as a concentration range from the concentration at which 50% of cancer cells survived with laser irradiation (therapeutic dose) to the concentration at which 80% of macrophage cells survived without laser irradiation (non-toxic dose) (Additional file 1: Fig S5). It should be noted that IBC-PB composites showed 84% cell viability even at the highest dose (2500 μg mL^−1^); accordingly, 2500 μg mL^−1^ was considered as a non-toxic dose of IBC-PB composites. We found that the in vitro therapeutic window for PB-NPs ranges from 76.7 to 97.7 ug ml^−1^, while for IBC-PB composites, it ranges from 72.7 to 2500 ug ml^−1^. Also, we defined the therapeutic index (TI) as the ratio between the therapeutic dose and non-toxic dose. The TIs for the PB-NPs and IBC-PB composites were 1.3 and 34.4, respectively.

### In vivo toxicity test

The biocompatibilities of the IBC-PB composites and PB NPs were assessed through a biochemical analysis 24 h after the subcutaneous injection in the right thighs and a blood draw of normal Balb/c-nude mice (n = 3) (Fig. 7c). The NS group served as a control group. The biocompatibility in each group was evaluated according to BUN, Cr, ALT, and AST, which are representative indicators of the kidney and liver function, respectively. In the BUN and Cr indices, the PB NPs and IBC-PB composites groups showed little difference numerically when compared with the NS group. The IBC-PB composites showed no significant difference compared to the NS group in terms of ALT and AST. However, in the PB NPs group, ALT was 1.30-fold higher and AST was 1.61-fold higher compared to that of the IBC-PB composites. The H&E-stained tissues from major organs (heart, liver, spleen, and kidney) in each group were imaged to confirm systemic toxicity (Fig. 7d). There were no significant signs of damage in any of the major organs, including the livers of the NS and IBC-PB composites groups. Meanwhile, prominent hepatocytic vacuolation was observed in the liver image of the PB NPs group, indicating acute liver toxicity [59, 60], unlike the liver tissue images of the other two groups. We speculate that for the IBC-PB composites, PB NPs were directly bound to BC, remaining at the injection site and not affecting the body, whereas the PB NPs were absorbed into the body 24 h after the injection and affected the liver function, leading to acute liver damage.

Several studies have described the use of engineered PB NPs to achieve efficient PTT effects, theranostic properties, or improved biocompatibility. Wang et al. describes the development of a boracic-acid-modified graphite carbon nitride and PB nanohybrid for theranostic applications, offering targeted Raman recognition and synergistic photothermal/photodynamic therapy in the near-infrared region [27]. A NaDyF4:50%Lu@PB nanocomposite was developed by Liu et al. as a biocompatible and effective PTT agent, which also possesses the ability to be used for magnetic resonance imaging [29]. Jing et al. developed a multifunctional nanoplatform by modifying hollow PB NPs with hyaluronic acid grafting polyethylene glycol and loaded with 10-hydroxycamptothecin for tumor-specific thermochemotherapy [61]. Chen et al. developed PEGylated PB NPs loaded with doxorubicin as a photothermal-chemo therapy for cancer [62]. Due to variations in therapeutic techniques, such as differences in laser settings and the use of additional therapeutic agents, it is challenging to make direct comparisons of therapeutic effects across studies. However, we were able to evaluate and compare the safety profile of the nanocomposites here, as presented in Table 1. Of the PB-containing nanocomposites, our IBC-PB composites exhibited the most biocompatible profile.

**Table 1:** Viability of cells treated with various forms of PB NPs

Particle name	Method	Cell viability at a certain concentration	Ref.
PB@Bg-C_3_N_4_	In situ reduction	95.6% at 200 μg mL^−1^	[27]
NaDyF_4_:50%Lu@PB	Solvothermal method	86% at 1000 μg mL^−1^	[29]
HCPT@HPBNs@PAA/PAH/HA-g-PEG	Self-etching reaction and PEGylation by ECD-NHS reaction	56% at 30 μg mL^−1^	[61]
PEGylated PB NPs	Precipitation, thin film hydration method	84.2% at 40 μg mL^−1^	[62]
IBC-PB composites	Trituration and self-etching reaction	83.59% at 2500 μg mL^−1^	Our study

*PB@ Bg-C*_*3*_*N*_*4*_ a boracic-acid-modified graphite carbon nitride and Prussian blue nanohybrid, *HCPT@HPBNs@PAA/PAH/HA-g-PEG* 10-hydroxycamptothecin loaded hyaluronic acid grafting polyethylene glycol modified hollow Prussian blue nanoparticles, *NaDyF*_*4*_*:50%Lu@PB* Prussian blue-coated NaDyF_4_ doped with Lu^3+^ ions nanocomposites, *PEGylated PB NPs* polyethylene glycol-attached Prussian blue nanoparticles

Various routes have been investigated for the administration of therapeutic nanoparticles, including intravenous, intramuscular, subcutaneous, intralesional, and perilesional types [63]. Among these routes, the localized administration of nanoparticles through intramuscular, subcutaneous, intralesional, and perilesional routes has been deemed ineffective for treating cancer due to the disseminating nature of cancer. However, several studies of cancer therapies based on locally injectable PB NPs have been reported, showing synergistic therapeutic effects when combined with other therapy strategies such as chemotherapy, radiotherapy, and immunotherapy. A gellan-based nanocomposite (NC) hydrogel embedding combretastatin A4 (CA4), a tubulin polymerization inhibitor for tumor growth suppression in cancer chemotherapy, and PB NPs showed a strong synergistic therapeutic effect as a type of NIR-triggered PTT and disrupting tumor vascular due to the continuous release of the CA4 [64]. An injectable PB-NPs-encapsulated agarose hydrogel, termed a PB reservoir and release controller (PRC) nanosystem, functioned as an improved combination therapy with PTT and radiotherapy (RT) given its excellent photothermal characteristics upon 808 nm NIR laser irradiation and with the catalytic capabilities of a radiosensitizer by converting endogenous hydrogen peroxide into oxygen for reactive oxygen species production by X-ray mediated RT [65]. A localized therapy using nanoparticles was also found to improve the efficacy of immunotherapy significantly, as the destruction of cancer cells in the nanoparticle-mediated therapy results in a release of cancer antigens, which in turn stimulates the adaptive immune system [66]. Juliana et al. used photothermal immunotherapy to enhance an immunotherapeutic effect with anti-cytotoxic T-lymphocyte antigen-4 (CTLA-4), well known as an immune checkpoint inhibitor (ICI), by means of PTT based on pH-dependent and intratumorally injectable PB NPs [67]. The studies discussed above suggest that IBC-PB composites could have broader applications, including disseminated cancer treatments, when used in combination with other cancer therapy systems, particularly immunotherapy.

## Conclusion

We developed a highly biocompatible, repeatable PTT agent by directly growing PB NPs on BC with a simple thermal reduction process. The synthesized IBC-PB composites had a monodispersed PB-NP-decorated BC morphology, a strong hyperthermal effect, an excellent PTT effect, and good recyclability. The IBC-PB composites showed a PTT effect similar to that of PB NPs but with a considerably higher safety profile compared to PB NPs. Consequently, the IBC-PB composites developed here is a promising nanomaterial based on PB that can function as a highly biocompatible and repeatable PTT agent.

## Experimental section

### Materials

Bacterial cellulose (nata de coco) was obtained from Vietnam Coco Food Co., Ltd. (Tang Nhon Phu, Vietnam). Potassium ferricyanide trihydrate (K_3_[Fe(CN)_6_]·3H_2_O), thiazolyl blue tetrazolium bromide, and Roswell Park Memorial Institute (RPMI) 1640 were obtained from Sigma-Aldrich (St. Louis, MO, USA). Cu(OH)_2_ was purchased from Alfa Aesar (Haverhill, MA, USA). The ammonia solution, sodium hydroxide (NaOH), hydrochloric acid (HCl), and diethyl ether used here were purchased from Samchun (Gangnam, Korea). Polyvinylpyrrolidone (PVP, K30) was purchased from Kanto Chemical Co. (Tokyo, Japan). Fetal bovine serum (FBS) was purchased from GE Healthcare Life Sciences (Buckinghamshire, UK), and 4% paraformaldehyde (PFA) was obtained from Biosesang (Seongnam, Korea) to fix the major organs. Female Balb/c nude mice (6–8 weeks) were purchased from Orient Bio (Seongnam, Korea) for the in vivo PTT experiment. The BD vacutainer^®^ SST^™^ system used in the study was purchased from BD Bioscience (New Jersey, USA).

### Preparation of PB NPs

PB NPs were synthesized according to a method reported in the literature [68]. PVP K30 (3 g) and K_3_[Fe(CN)_6_] (132 mg) were dissolved in a HCl solution (0.01 M, 40 mL) under vigorous magnetic stirring. After 30 min of stirring, a clear yellow solution was obtained, which was then placed in an oven at 80 ℃ for 24 h. The precipitates were collected by centrifugation and washed in distilled water, ethanol, and diethyl ether at 8000 rpm for 20 min several times. After drying at 60 ℃ in an oven for 24 h, PB nanoparticle powder was obtained.

### Synthesis of IBC

The impurities of nata de coco (1 kg) were removed by dialysis with deionized water (D.W.) for three days. After the washing process, the nata de coco was physically pulverized through a grinder and filtered through a sieve several times. Water was completely removed from the physically treated nata de coco through freeze-drying for more than 4 days. Schweitzer’s reagent was produced by dissolving copper hydroxide (100 mg) in an ammonia solution (5 ml) and stirring sufficiently. Lyophilized nata de coco (50 mg) was added to Schweitzer’s reagent (5 ml) and reacted for two hours at room temperature under vigorous stirring. Subsequently, mixture of Schweitzer’s reagent and bacterial cellulose was added to 20 ml of a 10% HCl solution for neutralization and the mixture was stirred sufficiently until the color no longer changed. The neutralized bacterial cellulose was filtered by a glass filter and washed with D.W. several times until the pH reached approximately 7. After washing, the collected bacterial cellulose was dispersed in D.W. (20 ml) and stored in a refrigerator at 5 ℃.

### Synthesis of IBC-PB composites

In 20 ml of the IBC solution, D.W. (70 ml), 1 M HCl (10 ml) and K_3_[Fe(CN)_6_]·3H_2_O (0.25 mmol, 0.5 mmol, 0.75 mmol, 1 mmol) were added, reaching a total solution amount of 100 ml. This mixture was reacted for 24 h at 80 ℃ oven. The IBC-PB composites were collected by filtration with a glass filter and washed with D.W. several times until the pH was approximately 7. After washing, the collected IBC-PB composites were dispersed in 50 ml D.W. and stored in a refrigerator at 5 ℃.

### Characterization of IBC-PB composites

The produced composites were characterized using a field-emission scanning electron microscope (FE-SEM, Hitachi S-4800). The crystal structure of the composites was confirmed by a powder X-ray diffraction analysis using a Bruker D8 Advance device (Cu Ka1 radiation, 5°min^−1^). A thermogravimetric analysis (TGA) was conducted using a TGA/DSC 1 analyzer (Mettler Toledo) with a heating rate of 5 ℃ min^−1^ in air. An inductively coupled plasma atomic emission spectroscopy (ICP-AES) analysis was conducted in 6000 K Ar plasma with a range of 167–782 nm (OPTIMA 8300, Perkin-Elmer, USA). The absorbance was measured by a microplate reader (SYNERGY H1, BioTek, Winooski, VT, USA). An 808 nm NIR laser (FC-W-808-10W, CNI, Changchun, China) was used for photothermal imaging and PTT studies. A thermal imaging camera (HT-18, HT Instruments, Faenza, Italy) was used for real-time hyperthermal imaging. A blood biochemistry analyzer (Hitachi 7020, Tokyo, Japan) was utilized for the blood biochemical analysis. A Nicolet5700 FTIR spectrometer (Thermo electron Corporation, United States) with a measurement range of 4000–400 cm^-1^ was used to record the infrared spectra.

### Photothermal performance test

The IBC-PB composites (0.2 mg 1.5 ml^−1^) dispersed in D.W. was added to a 4 ml vial, a stirring bar was added, and the surroundings were wrapped with Styrofoam. An 808 nm NIR laser (1 W cm^−2^) was irradiated 5 mins onto each sample and the temperature was measured every 30 s. According to the experimental results, we collected the best sample, and it was used for a retention test. The retention test was conducted by repeating the 5 min laser irradiation, which was followed by cooling to the initial temperature 5 times.

### The evaluation of in vitro cytotoxicity and photothermal therapeutic effect

4T1 breast cancer cell and RAW 264.7 macrophage cell lines were authenticated and obtained from the Korean Cell Line Bank, Korea). The cells were cultured, added to a 96-well microplate (10^3^ cells 200 μL^−1^ of RPMI 1640 or DMEM), and incubated at 37 ℃ with 5% CO_2_ for 24 h. After incubation, the cell media were discarded and the cells were washed with DPBS three times to remove remained media, followed by an exchange for new cell media in each well. The PB NPs and IBC-PB composites were incubated with RAW 264.7 cells with PB concentration gradients (0, 25, 50, 100, 250, 500, 1000, and 2500 μg mL^−1^). The BC and IBC-PB composites were added to the 4T1 cancer cells with the series of BC concentrations (0, 10, 50, 100, 200, and 500 μg mL^−1^). All nanomaterial treated cells were incubated overnight. The IBC-PB composite-treated 4T1 cells were irradiated with an 808-nm laser at 2 W cm^−2^ for five minutes and another 24 h of incubation was conducted. Subsequently, 0.5 mg mL^−1^ of MTT solution was added to each well after incubation and a DPBS washing step for two hours for the MTT assay. After the incubation of the MTT solution, cell images were obtained by an optical microscope of the IBC-PB composite-treated groups before the DMSO solvent exchange step. Each well was measured at 540 nm by the microplate reader to acquire its absorbance. Cell viability was calculated at a ratio relative to untreated control samples and the data were evaluated by a t-test. In vitro cytotoxicity test was carried out in triplicate.

### Preparation of the 4T1 tumor-bearing mouse model

The 4T1 cancer cells were cultured and collected with PBS (10^5^ cells 15 μL^−1^ PBS). The condensed 4T1 cells were injected subcutaneously into the right thigh of female Balb/c nude mouse. Further in vivo experiments with the mouse tumor model were performed when the tumor size reached approximately 50–100 mm^3^. All animal studies were approved by the Institutional Animal Care and Use Committee of Woojung Bio Inc.

### In vivo photothermal imaging

Normal saline (NS), the PB NPs, and the IBC-PB composites were used for a peritumoral injection (p.i.) at the tumor region in the 4T1 tumor bearing mice. After each injection, an 808 nm laser was irradiated at 2 W cm^−2^ for 5 mins, in vivo photothermal imaging was conducted, and photothermal images were obtained in each group during laser irradiation.

### In vivo photothermal therapy study

First of all, the mice were randomized into three groups. The NS, PB NPs, and IBC-PB composites underwent a p.i. procedure around the tumor region in the 4T1 tumor bearing mice (n = 3). The 808 nm laser was irradiated under a condition identical to that used for the in vivo photothermal imaging step after the injection. The treated and untreated groups underwent a follow-up assessment for 18 days that involved measuring the tumor sizes and weights. The data were evaluated statistically by an ANOVA test.

### The assessment for tumor retention of in vivo PB NPs and IBC-PB composites

The PB NPs and IBC-PB composites were inserted by peritumoral injection at the 4T1 tumor region. Photographic images were obtained at various time points post injection (0, 1, and 7 days). The tumor tissues were resectioned and internal photographic images were obtained.

### Biocompatibility test of IBC-PB composite and PB NPs

The NS, PB NPs, and IBC-PB composites were subcutaneously injected into the right thigh region in each case. Blood samples with a volume of 500 μL were collected with a SST vacutainer in each group 24 h after the injection. The obtained blood samples were centrifuged, and the supernatant was re-collected for each sample. The supernatant was analyzed for indices that represent liver and kidney functions (alanine transaminase (ALT), aspartate transaminase (AST), creatinine (Cr), and blood urea nitrogen (BUN)) by a blood biochemistry analyzer. After the blood draw, major organs in each group were collected, stored in a 4% PFA solution, stained with hematoxylin and eosin (H&E) staining, and paraffin-sectioned for tissue imaging.

### Supplementary Information

**Additional file 1: Figure S1.** SEM image of the bacterial cellulose (BC) to verify the morphology and structure. **Figure S2.** SEM image of the PB NPs to verify the size and morphology. **FigureS3.** FTIR spectra for BC and IBC-PB composites. **Figure S4.** Photographic images of in vivo retention ability comparison between PB NPs and IBC-PB composites at different time points (0, 1, 7 days). White circle indicate the peritumoral injected IBC-PB composites. **Figure S5.** In vitro therapeutic window determination of PB NPs and IBC-PB composites. (a) therapeutic dose and (b) non-toxic dose of PB NPs, and (c) therapeutic dose and (d) non-toxic dose of IBC-PB composites.

## Data Availability

The datasets used and/or analysed during the current study available from the corresponding author on reasonable request.
