# Supplementary material for: Injectable biocompatible nanocomposites of Prussian blue nanoparticles and bacterial cellulose as a safe and effective photothermal cancer therapy
Source: J Nanobiotechnology. 2023 Oct 5;21:365. doi: 10.1186/s12951-023-02108-6 (PMC10552393; doi:10.1186/s12951-023-02108-6)
Supplement: Supplementary file 1 — Additional file 1: Figure S1. SEM image of the bacterial cellulose (BC) to verify the morphology and structure. Figure S2. SEM image of the PB NPs to verify the size and morphology. FigureS3. FTIR spectra for BC and IBC-PB composites. Figure S4. Photographic images of in vivo retention ability comparison between PB NPs and IBC-PB composites at different time points (0, 1, 7 days). White circle indicate the peritumoral injected IBC-PB composites. Figure S5. In vitro therapeutic window determination of PB NPs and IBC-PB composites. (a) therapeutic dose and (b) non-toxic dose of PB NPs, and (c) therapeutic dose and (d) non-toxic dose of IBC-PB composites. [file 12951_2023_2108_MOESM1_ESM.pptx]

## Slide 1
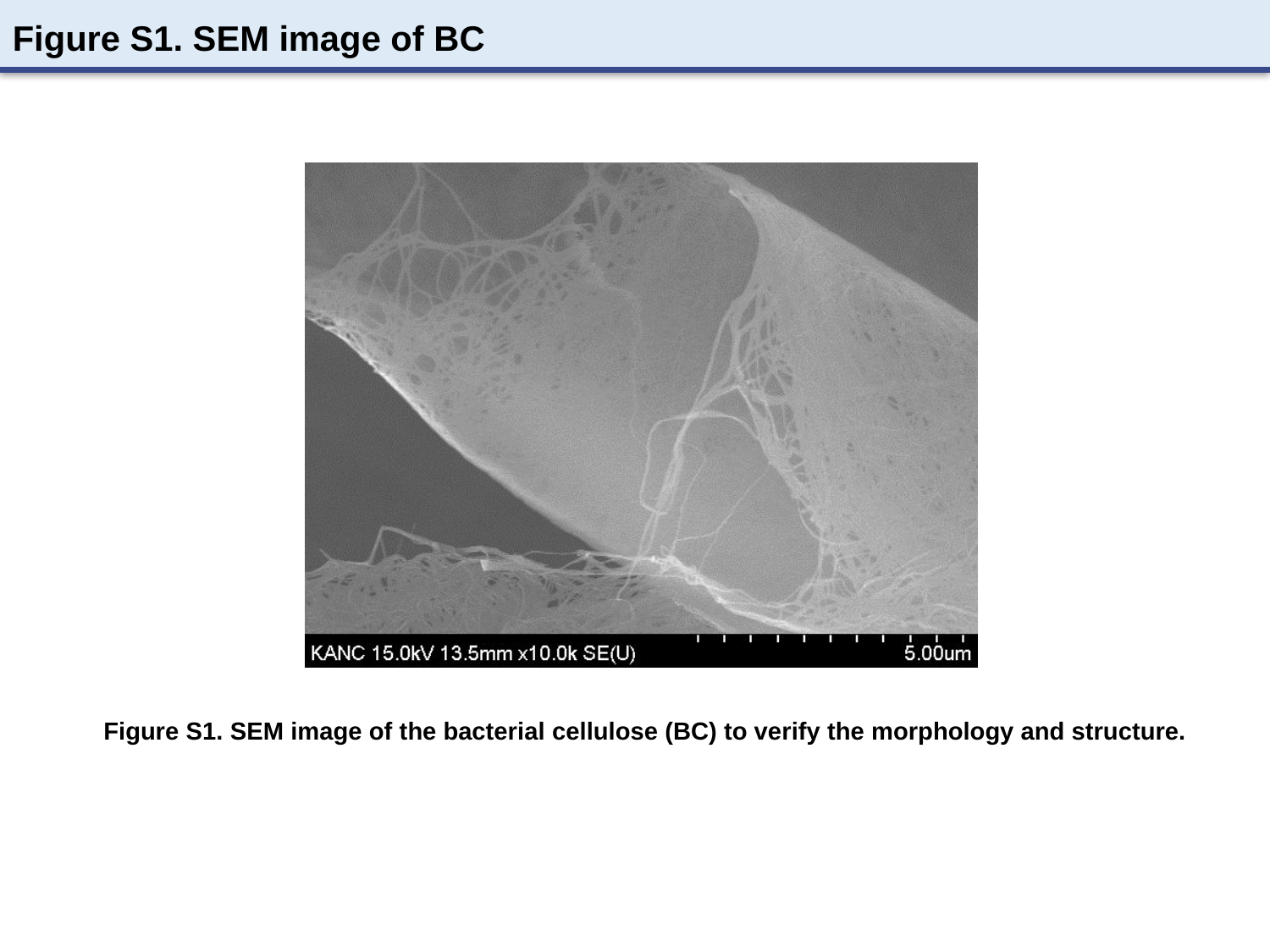

Figure S1. SEM image of BC
Figure S1. SEM image of the bacterial cellulose (BC) to verify the morphology and structure.

## Slide 2
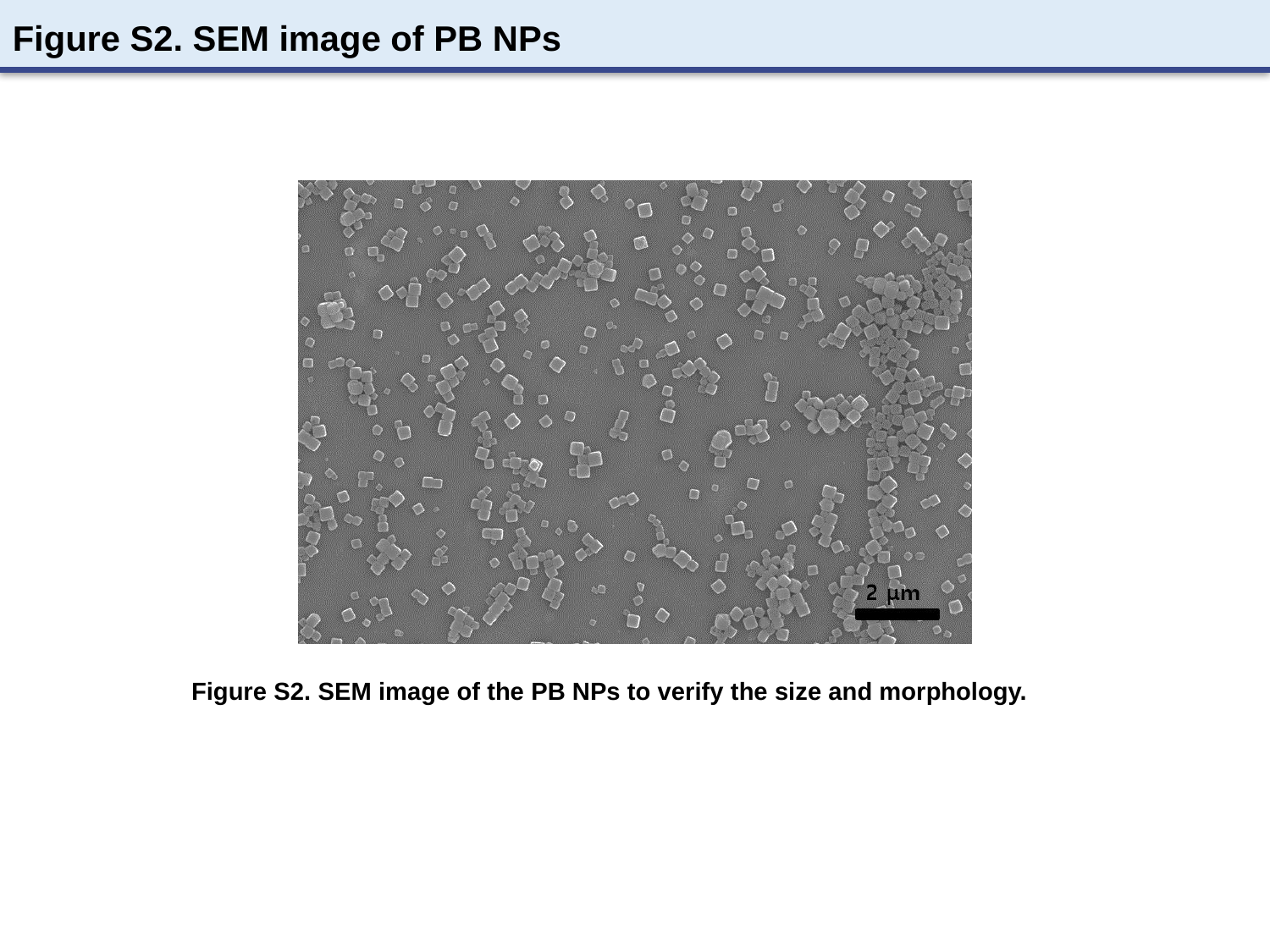

Figure S2. SEM image of PB NPs
Figure S2. SEM image of the PB NPs to verify the size and morphology.

## Slide 3
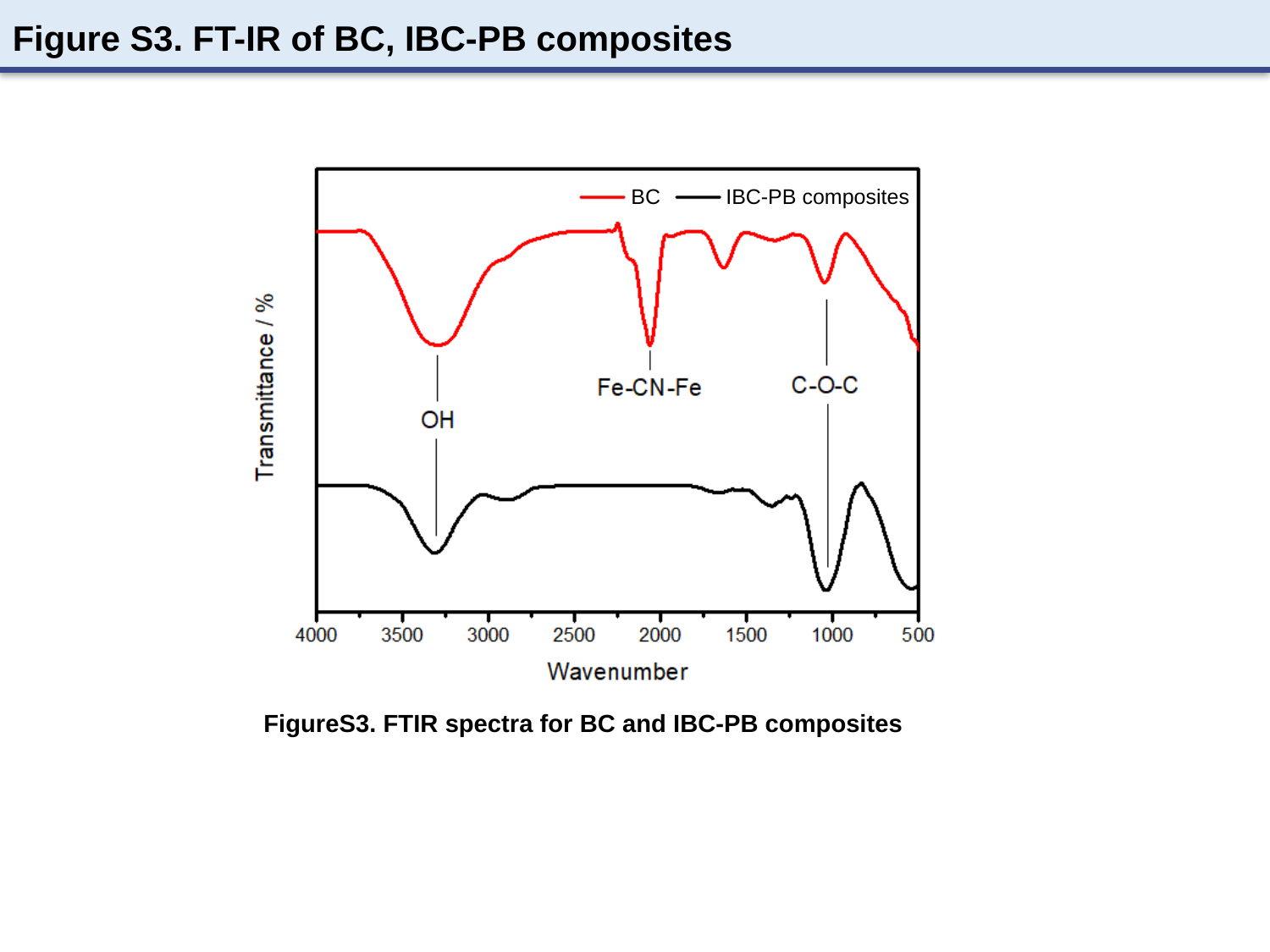

Figure S3. FT-IR of BC, IBC-PB composites
BC
IBC-PB composites
FigureS3. FTIR spectra for BC and IBC-PB composites

## Slide 4
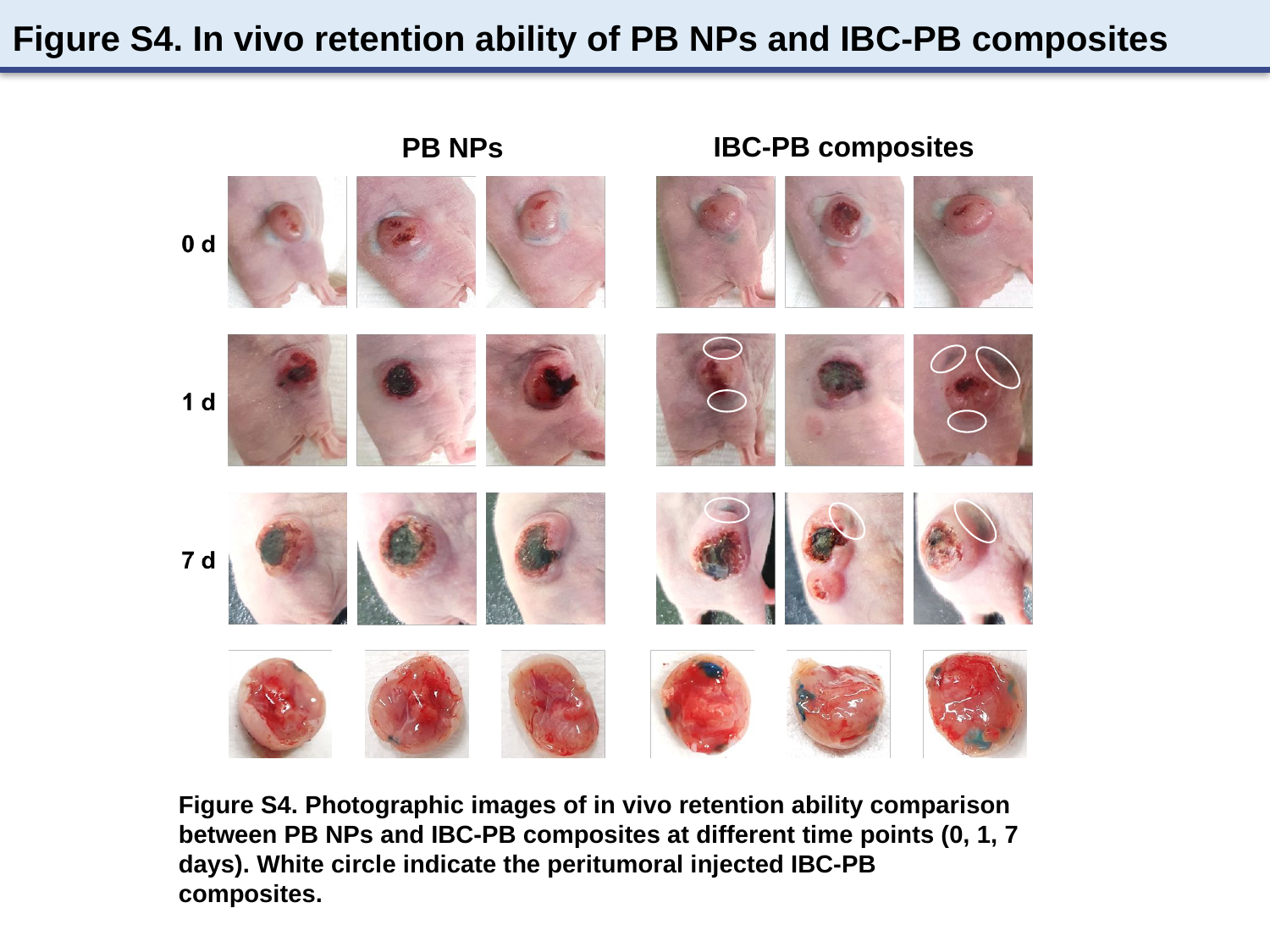

Figure S4. In vivo retention ability of PB NPs and IBC-PB composites
IBC-PB composites
PB NPs
Figure S4. Photographic images of in vivo retention ability comparison between PB NPs and IBC-PB composites at different time points (0, 1, 7 days). White circle indicate the peritumoral injected IBC-PB composites.

## Slide 5
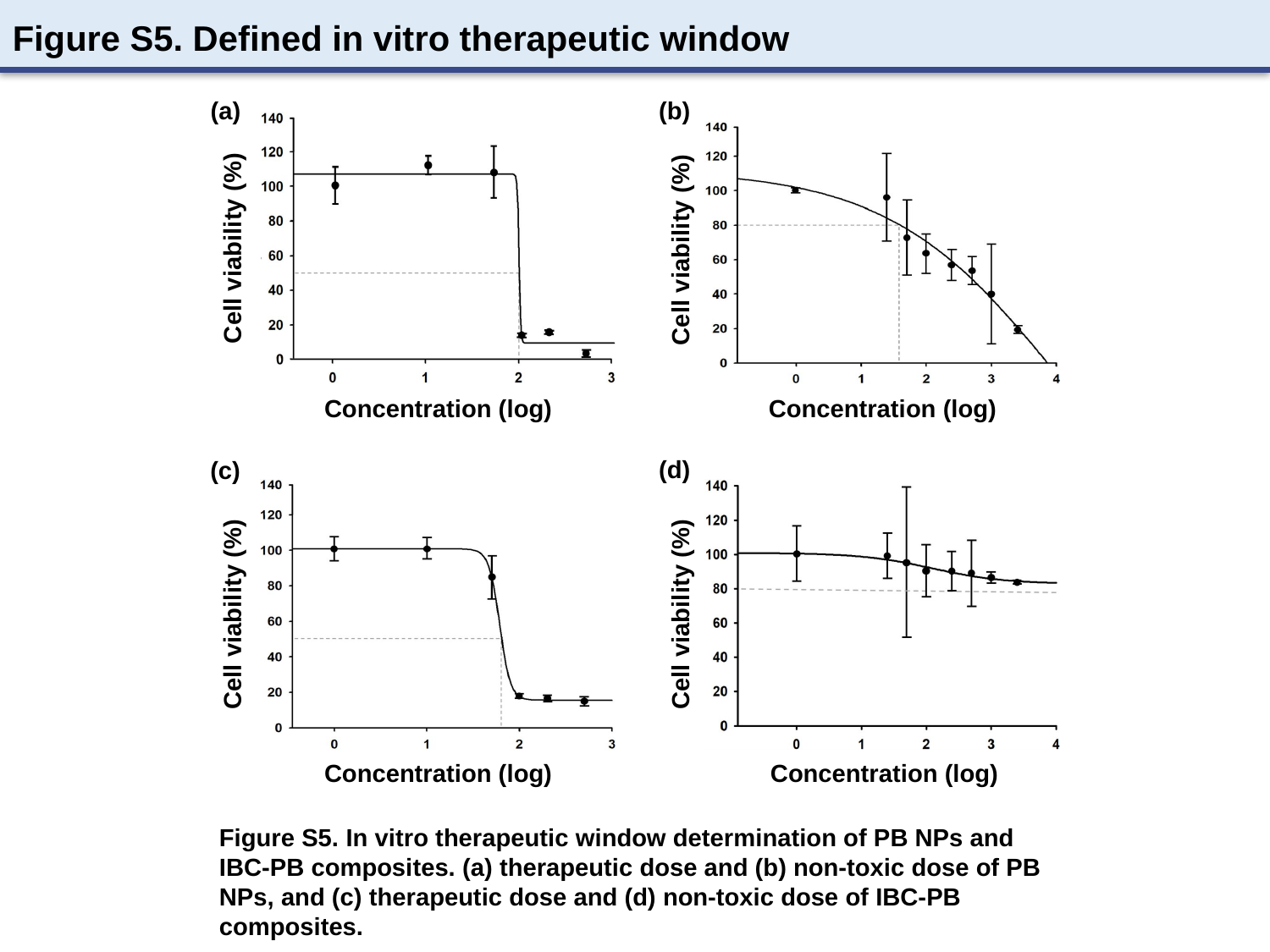

Figure S5. Defined in vitro therapeutic window
(b)
(a)
Cell viability (%)
Cell viability (%)
Concentration (log)
Concentration (log)
(d)
(c)
Cell viability (%)
Cell viability (%)
Concentration (log)
Concentration (log)
Figure S5. In vitro therapeutic window determination of PB NPs and IBC-PB composites. (a) therapeutic dose and (b) non-toxic dose of PB NPs, and (c) therapeutic dose and (d) non-toxic dose of IBC-PB composites.
